# Supplementary material for: Using an In Vivo Mouse Model to Determine the Exclusion Criteria of Preexisting Anti-AAV9 Neutralizing Antibody Titer of Pompe Disease Patients in Clinical Trials
Source: Viruses. 2024 Mar 5;16(3):400. doi: 10.3390/v16030400 (PMC10976115; doi:10.3390/v16030400)
Supplement: Supplementary file 1 [file viruses-16-00400-s001.zip › viruses-2787684-supplementary.pdf]

viruses

Using *in vivo* mouse model to determine the exclusion criteria of preexisting anti-AAV9 neutralizing antibody titer of Pompe disease patients in clinical trials

HanQing WANG [1], CengCeng Zhang [1], ZheYue Dong [2], XueYang Zhu[1]，XuChu Zheng[1]，Ziyang Liu[1]，JianFang Zhou[1]，Shuangqing Yu[1*]，Xiaobing Wu[1*]，Xiaoyan Dong[1]

[1] Genecradle Therapeutics Inc, Beijing, China, 100176; wuxiaobing@bj-genecradle.com
[2] Beijing FivePlus Gene Technology Co. Ltd., Beijing, China 102629; dongzy@fiveplusgene.com.cn
* Correspondence: yushuangqing@bj-genecradle.com; wuxiaobing@bj-genecradle.com

Supplementary information

Efficient transductions of GC301 in heart and liver in a comparable neutralizing level of human IVIG-pretreated mice at high dosage

Since total neutralizing titer of polyclonal antibodies in serum is usually measured in practice, we assayed the effects of human IVIG to see whether the preclusion criteria, NAb titers >1:100 in our study, is suitable for future clinical trial. The animal experiment was conducted as demonstrated in Figure S1. We used a high dose of GC301, 1.2E+14 vg/kg to identify the effects of pre-existing NAbs on transduction efficiency. The serum before injection of GC301 were collected for anti-AAV9 NAb testing. A comparable neutralizing level of human IVIG, 1:61 (1:47~1:78) was obtained, as that of 1.5μg/ml anti-AAV9 MoAb, 1:87 (1:78~1:99) (Table S1). Subsequently, we compared the transductions of GC301 in presence of the low level of pre-existing NAb, IVIG and 1.5 μg/ml anti-AAV9 MoAb on Day28 post injection. We discovered that the vector DNA copies present in heart in presence of IVIG and 1.5 μg/ml anti-AAV9 MoAb, (Figure 2S, A) was around 61% and 78% of the no pretreated mice, respectively (89,279±18,576 *vs* 146,807±64,054 copies/μg DNA; 116,421±26,589 *vs* 146,807±64,054 copies/μg DNA) while the GAA activity in heart is around 85% and 135% of no pretreated mice(4,317±634 *vs* 5,052±1,307 nmol/mg/h; 6,840±723 vs 5,052±1,307 nmol/mg/h) (Figure 2S,C). Furthermore, both a comparable neutralizing level of human IVIG and anti-AAV9 MoAb, as <1:100 have little effect on transduction efficiency of GC301 in liver as compared with those of no pretreated mice on Day28 at 1.2E+14 VG/kg (Figure2S, D, 1,426±187.18 *vs* 1,603±212.29 nmol/mg/h; 1,627±353.61 *vs* 1,603±212.29 nmol/mg/h). There were no statistically significant differences among all GC301 infused groups. Thus, the criteria of preexisting AAV9 NAbs could be adopted in future clinical trial.

.

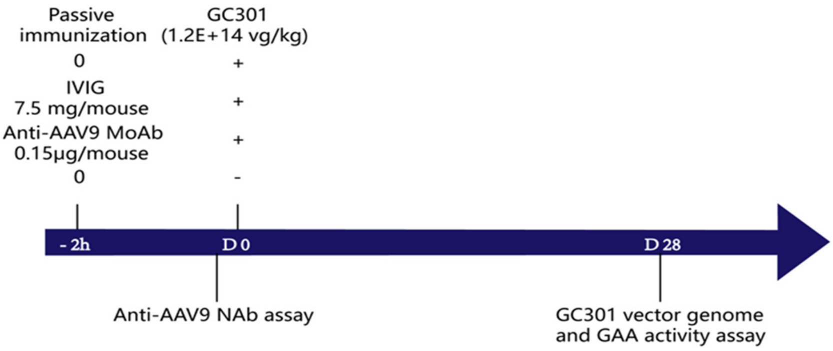

Figure S1. Experimental protocol for GC301. Female Balb/C mice aged 4~6 weeks were used and randomly assigned to each group, 5 animals in each group. Animals were passively immunized by tail vein injection with 7.5 mg human purified immunoglobulin (IVIG) or 0.15μg anti-AAV9 MoAb in final volume of 100μl and mice with no Ab-treatment denoted as 0 and those injected with PBS were used as negative control (NC). After 2hr, the sera were collected for anti-AAV9 NAb testing and then the mice received 1.2E+14 vg/kg of GC301 by tail vein route, mice were sacrificed on Day28 post GC301 injection, GAA activity and GC301 vector genome in heart and liver were assayed.

Table S1. The NAb titers of passively immunized mice.

Group	Geometric mean of passively immunized mice sera[1] (95% CI)
0	<1:20
IVIG	1:61 (1:47~1:78)
1.5	1:87 (1:77~1:99)
NC	<1:20

[1] The sera were collected 2hr after infusion with 100μL mice negative serum (denoted as 0), 1.5 μg/ml MoAb in mice negative serum (denoted as 1.5) or IVIG. Mice with no Ab-treatment denoted as 0 and those injected with PBS were used as negative control (NC). N=5 per group.

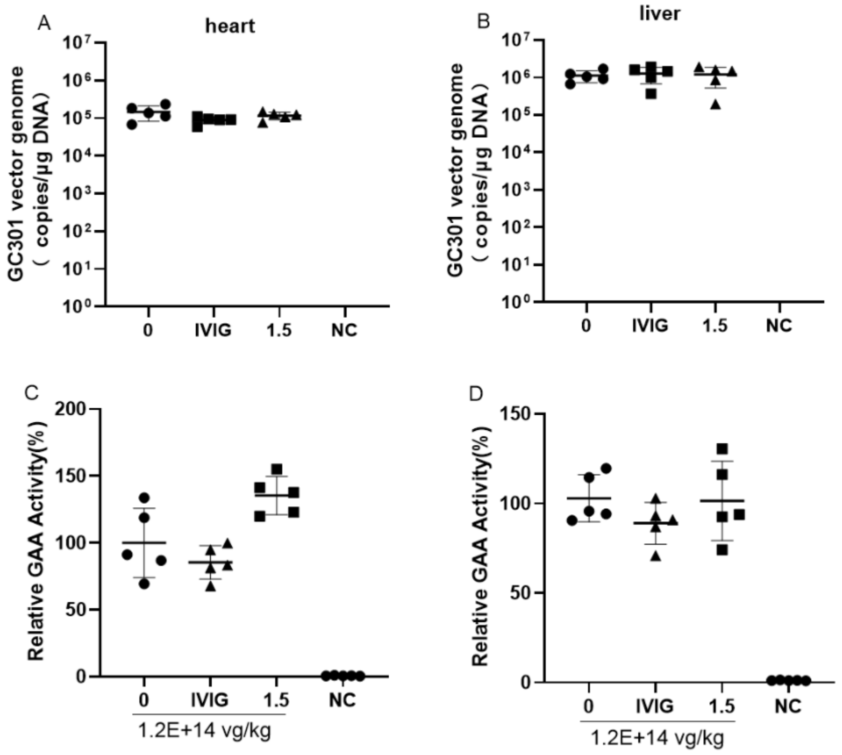

Figure S2. Vector genome DNA copies and GAA activity in the heart and liver of Balb/c mice post rAAV9-coGAA (GC301) injection. Mice were injected with rAAV9-coGAA (GC301) at the dose of 1.2E+14vg/kg, n=5. The vector DNA copies in heart (A) and liver (B) on Day28 post GC301 injection. The relative GAA activity in heart (C) and liver (D) on Day28 post GC301 injection.

Disclaimer/Publisher's Note: The statements, opinions and data contained in all publications are solely those of the individual author(s) and contributor(s) and not of MDPI and/or the editor(s). MDPI and/or the editor(s) disclaim responsibility for any injury to people or property resulting from any ideas, methods, instructions or products referred to in the content.
